# STaRT-RWE: structured template for planning and reporting on the implementation of real world evidence studies

**DOI:** 10.1136/bmj.m4856

**Published:** 2021-01-12

**Authors:** Shirley V Wang, Simone Pinheiro, Wei Hua, Peter Arlett, Yoshiaki Uyama, Jesse A Berlin, Dorothee B Bartels, Kristijan H Kahler, Lily G Bessette, Sebastian Schneeweiss

**Affiliations:** 1 Division of Pharmacoepidemiology and Pharmacoeconomics, Department of Medicine, Brigham and Women’s Hospital and Harvard Medical School, Boston, MA, USA; 2 Division of Epidemiology, Office of Surveillance and Epidemiology, Food and Drug Administration, Silver Spring, MD, USA; 3 Data Analytics and Methods Taskforce, European Medicines Agency, London, UK; 4 Faculty of Epidemiology and Population Health, London School of Hygiene and Tropical Medicine, London, UK; 5 Office of Medical Informatics and Epidemiology, Pharmaceuticals and Medical Devices Agency, Tokyo, Japan; 6 Johnson and Johnson, Titusville, NJ, USA; 7 UCB Pharma, Brussels, Belgium; 8 Hannover Medical School, Hannover, Germany; 9 Novartis Pharmaceuticals Corporation, East Hanover, NJ, USA

## Abstract

In alignment with the International Council of Harmonization’s strategic goals, a public-private consortium has developed a structured template for planning and reporting on the implementation of real world evidence (RWE) studies of the safety and effectiveness of treatments. The template serves as a guiding tool for designing and conducting reproducible RWE studies; set clear expectations for transparent communication of RWE methods; reduce misinterpretation of prose that lacks specificity; allow reviewers to quickly orient and find key information; and facilitate reproducibility, validity assessment, and evidence synthesis. The template is intended for use with studies of the effectiveness and safety of medical products and is compatible with multiple study designs, data sources, reporting guidelines, checklists, and bias assessment tools.

Real world evidence (RWE) generated from sources of real world data via the application of principled database epidemiology increasingly informs important decisions about the clinical effectiveness of medical products and interventions.^1^ ^2^ ^3^ ^4^ ^5^ Unlike clinical trials, which can leverage the power of randomisation, or non-randomised studies with prospective data collection for a specific research purpose, most RWE studies make secondary use of electronic data collected as part of routine healthcare processes (eg, administrative claims and electronic health records). Generating high quality evidence when analysing data not collected for research purposes requires decision making about many complex design and analytical parameters to handle temporality, measurement, confounding, and other potential sources of bias. Compared with trials and non-experimental studies that prospectively collect data for a research question, RWE studies have greater variability in design and analysis options. Owing to the current lack of structure in study reporting, assessment of RWE studies often requires substantial resources within regulatory and other organisations.

Despite recommendations from the International Committee of Medical Journal Editors that the methods sections of research publications should provide enough detail so that others with access to the data would be able to reproduce the results,^6^ attempts to replicate results from database studies have been hampered by a lack of clarity in reporting on critical study implementation details.^7^ ^8^ ^9^ ^10^ ^11^ Many organisations recognise this problem and have created guidelines and checklists for research reporting.^12^ ^13^ ^14^ ^15^ ^16^ ^17^ ^18^ ^19^ ^20^ ^21^ ^22^ Existing guidelines and checklists already have a strong consensus regarding what main elements are important to report. However, these guidelines are general in order to cover a broad base—which leaves room for ambiguity, assumptions, and misinterpretation when planning and implementing RWE studies.^7^ ^8^

The multidisciplinary, multidatabase, and collaborative nature of RWE study design and conduct would be improved by clearer communication of critically important details. This need is particularly relevant for common protocol studies involving collaboration between multiple groups, where different interpretation by the groups executing a protocol can substantially influence results.^23^ Unambiguous documentation of a research team’s intended study implementation parameters can increase the effectiveness of communication within the multidisciplinary study team as well as between research teams and decision makers reviewing their studies.

Ambiguity in communication of key design and analytical details as well as data sources and their origins makes it difficult for reviewers to assess potential for bias or evaluate the robustness of study findings, and could limit the use of RWE studies in regulatory and other healthcare decision making.^24^ Recognising the high variability in quality and completeness of communication about RWE study implementation,^7^ ^8^ an increasing number of stakeholders have moved towards routine pre-registration of RWE studies with fully specified protocols to support regulatory and coverage decisions.^25^ The International Council for Harmonization has set short term strategic goals of harmonising the structure and format of protocols and reporting documents in regulatory submissions to increase the acceptability of RWE studies.

Regulators are increasingly calling for high levels of transparency as an integral part of the science of RWE. Although the need for transparent and reproducible evidence was recognised earlier, the coronavirus disease 2019 pandemic has brought the field to an inflection point. Currently, timely high quality evidence is urgently needed to inform decision making. Such evidence can be obtained through rigorous analysis of routinely collected healthcare data. However, an influx of high profile studies with methodological issues has negatively affected the credibility of evidence derived from analysis of non-randomised healthcare databases. In order to have a foundation from which to evaluate and distinguish useful, rigorously designed studies from studies with validity problems, regulators and other decision makers have called for researchers to conduct and report RWE studies using standards agreed on by professional societies dedicated to RWE. Based on best scientific practice,^26^ this tool aims to resolve the identified gap in transparency on RWE study methods.

**Summary points**

Compared with clinical trials and non-experimental studies that prospectively collect data, studies that use routinely collected electronic healthcare data have a greater variability in design and analysis options
Existing guidelines and checklists have a strong consensus regarding what main elements are important to report, but they can lead to ambiguity, assumptions, and misinterpretation when planning and implementing RWE studies
An increasing number of stakeholders have moved towards routine registration of RWE studies with fully specified study implementation protocols to support regulatory and coverage decisions
Through a public-private collaboration with broad and international stakeholder input, a structured template for planning and reporting on RWE study implementation (STaRT-RWE) has been developed
STaRT-RWE is intended to serve as a didactic tool for designing and conducting good RWE studies; set clear expectations for communication of RWE methods; reduce misinterpretation of prose that lacks specificity; allow reviewers to quickly find key information; and facilitate reproducibility, validity assessment, and evidence synthesis
The template has been endorsed by the International Society of Pharmacoepidemiology (ISPE) and the Transparency Initiative led by the International Society of Pharmacoeconomics and Outcomes Research in partnership with ISPE, Duke Margolis Health Policy, and the National Pharmaceutical Council

## Structured template and reporting tool for real world evidence (STaRT-RWE)

While the simplicity of a checklist is ideal for summarising areas to report, it leaves room for misinterpretation and ambiguity about important details of study implementation. We complement the checklist approach by developing a study implementation template where methods related items from existing checklists correspond to the main headings in structured tables where critical details are communicated. Design details are visually summarised in a figure based on a framework for graphical depiction of longitudinal study design.^27^ The template is intended to support both research planning and communication of methods. As such, it should be completed when conceptualising and designing a study, updated with version changes during implementation, and then shared with the final study results at the time of submission to enable review and replication. The template tables can be referenced if they were previously used in study registration materials or publicly posted. Alternatively, they can be published as supplemental material. Used in this way, the template will increase the rigor of study conduct as well as support understanding of methods and interpretation of results by decision makers.

The study implementation template is designed to fulfil several aims: serve as a guiding tool for designing and conducting reproducible RWE studies; set clear expectations for transparent communication of RWE methods; reduce misinterpretation of prose that lacks specificity; allow reviewers to quickly orient and find key information; and facilitate reproducibility, validity assessment, and evidence synthesis. This multipurpose tool is particularly relevant for hypothesis evaluating studies on treatment effectiveness and safety^28^ that are intended to influence a regulatory or coverage decision, but is also compatible with RWE studies of various types (eg, exploratory, descriptive, prediction).

## Development of a template for planning and reporting on RWE study implementation

The detailed study parameters requested in the structured template on study implementation are from a consensus document developed by a joint task force between the International Society for Pharmacoepidemiology (ISPE) and International Society for Pharmacoeconomics and Outcomes Research (ISPOR).^15^ The consensus document includes a detailed catalogue of specific parameters that represent key scientific and operational decisions made when implementing database studies to facilitate reproduction, facilitate replication, as well as evaluate validity. Many of these parameters are incorporated into existing reporting checklists at a high summary level, 14 19 as areas which researchers should provide detail on. However, the type and nature of detail have been explicitly delineated in the STaRT-RWE template for study implementation.

**Fig 1:**
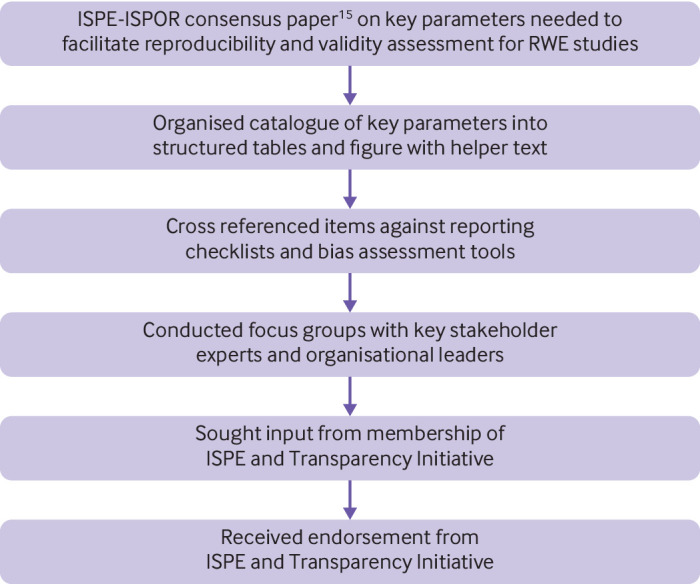
Development of template for planning and reporting on real world evidence (RWE) study implementation.^15^ ISPE=International Society of Pharmacoepidemiology; ISPOR=International Society of Pharmacoeconomics and Outcomes Research; Transparency Initiative=multistakeholder group led by ISPOR in partnership with ISPE, Duke Margolis Health Policy, and National Pharmaceutical Council

We cross referenced the catalogue in the ISPE-ISPOR consensus document against the design and analysis sections of existing reporting checklists for non-experimental studies such as RECORD-PE^14^ and ENCePP^19^ as well as against published quality assessment tools^29^ ^30^ ^31^ to ensure that key elements for assessing validity were covered (fig 1). We then created a series of structured tables detailing these study implementation parameters, accompanied by graphical depiction of longitudinal study design.^27^ The STaRT-RWE template tries to strike a balance between modularised response options while retaining flexibility. Prose is limited in the structured template tables, therefore the structured tables detailing study parameters should be accompanied by prose based background and rationale for scientific decisions, as well as interpretation of results and discussion in manuscripts or reports. Unlike reporting checklists, which have a more limited set of items that are requested somewhere in a paper, STaRT-RWE helps investigators walk through their intended scientific decisions in organised, unambiguous detail when planning their study. This implementation plan can then made available in online appendices when reporting on the completed study.

## STaRT-RWE template tables and figure 

In close alignment with the detailed elements identified in the joint ISPE-ISPOR task force consensus document, the template includes several tables that are detailed below (appendix 1). We have included the template tables with example entries (highlighted in yellow) to show how the tables could be completed for a simple, comparative cohort study of a time-to-event outcome in pharmacoepidemiology. In practice, the template table entries would depend on the design and analysis parameters selected for the study. Detailed instructions integrated in the header text of the template tables can be deleted after the table is populated.

### Administrative information

Administrative information is summarised in the first three tables of the template (appendix 1), including a table of contents, the study title, primary and secondary PICOT objectives (population, intervention, comparator, outcome, and time horizon), other administrative details as appropriate, and a version history documenting not only what changed, but also why it was changed, and when the change was made—documenting the history of the planned analyses.

### Study design

**Fig 2:**
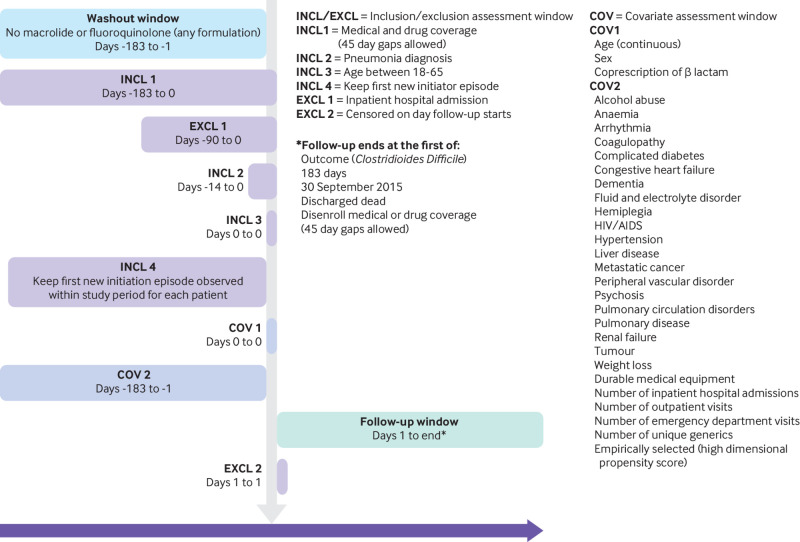
Example study design diagram comparing use of macrolide versus fluoroquinolone on *Clostridioides Difficile*

The study design diagram (fig 2) provides a concise summary of how the analytical cohort was created. It is read from top to bottom, showing the sequence of actions taken to create the cohort. The vertical arrow denotes day 0, the point at which the study entry defining criterion is met. The size and location of the horizontal bars visually show the temporality of assessment windows for inclusion or exclusion criteria, covariates, and follow-up relative to day 0, while number ranges within each bar use standard mathematical notation to explicitly denote the temporality. The diagram can also have footnotes providing high level details about what is defined within each of the assessment windows. Details were published earlier this year, along with Microsoft PowerPoint templates.^27^

### Study population

The study population parameter table (appendix table 3) provides more detail than the design diagram, starting with metadata about the data source, any data linkages, and name or version of software used to create the analytical cohort. In subsection A of appendix table 3, the data source name and version are identified, as well as any sampling criteria applied (eg, the data cut only includes patients with a diagnosis of diabetes). If data linkage was involved, the table has a space to provide a citation or refer to an appendix with description of the linkage (eg, how it was done, performance characteristics).

### Remaining template sections

The remaining subsections of this table detail, in a structured way, the operational definitions for the index date for cohort entry, exposures, inclusion or exclusion criteria, and order of application, covariates, outcomes, and follow-up time (appendix table 3B-H). These subsections summarise what is measured, timing of measurement, care setting (eg, inpatient, outpatient, emergency department), type of codes used to define the measure (eg, drug, diagnosis, procedure or laboratory codes), as well as sources for the algorithms (eg, publication, clinician review). For algorithms based on diagnosis codes, the template includes a section for researchers to define whether codes are required to be in the primary position (indicating that the diagnosis is the main reason for the encounter). The actual clinical codes used to define each measure are specified in appendices to the template tables; these appendices are structured and machine readable.

A subsection (appendix table 3B) focuses on detailing the day 0 defining criteria for entry to the study population. This subsection includes fields to specify how many times patients can enter the cohort, the type of entry, washout windows for incident entry if relevant, and what defines incident entry (eg, new users of a drug or newly diagnosed with a particular condition). The subsections on inclusion and exclusion criteria (appendix table 3C-D) have dropdown fields for specifying whether the criteria are applied before or after selection of the date for entry to the analytic study population. For covariates (appendix table 3E), how the variable is operationalised is also defined (eg, continuous, binary, categorical), with the option to provide more detail about transformations. The template includes an optional section on empirically defined covariates (appendix table 3F), where the source code or reference for the algorithm used for data driven selection of covariates are provided (eg, high dimensional propensity score algorithm^32^ ^33^). Throughout the table, fields indicate whether the study parameter was prespecified and whether it was varied in sensitivity analyses.

In the outcome subsection (appendix table 3G), the primary outcome is specified along with measurement characteristics. Outcome measurement performance could come from published algorithm validation papers, from direct validation of outcomes within the analytical cohort, or researchers could explicitly note that there is no information about the accuracy of outcome ascertainment. The type of outcome and related washout windows to define incidence if relevant, care settings, and diagnosis positions are also specified in this section.

In the follow-up subsection (appendix table 3H), investigators define when follow-up begins relative to cohort entry and how it ends, which can guide investigators to consider each option and make an active choice. It also makes clear for the reviewer what are and are not used as censoring criteria. If censoring on discontinuation is selected, the table includes sections to define duration of exposure, including a grace period to allow for non-adherence, to define whether or how an algorithm is applied to handle early refills, or build in an induction window for the hypothesised biologic window of effect of exposure or carry over effects for the effect of a drug beyond the end of exposure.

### Additional tables

Analysis specifications are provided in a separate table (appendix table 4), including fields for the hypothesis being tested and study population for the primary analysis and relevant subgroup analyses, followed by the same information for secondary analyses in subsequent sections. The software packages can be reported here, the models that are fit, the type of confounding adjustment and variables included, with specification of some parameters such as matching ratio and caliper, formulas for weights, trimming, and truncation rules. This table also includes fields to specify how missing data are handled in the analysis and subgroup analyses (eg, excluding patients with missing or unknown sex and multiple imputation for missing total cholesterol values).

A separate table for sensitivity analyses (appendix table 5) also exists, where investigators can specify which parameters are being changed, provide rationale for why they are being varied and detail what investigators expect to learn from this sensitivity analysis compared to the primary analysis.

Additional study population table shells that suggest the presentation of findings are provided (appendix tables 6 and 7), including an attrition table showing the counts as inclusion and exclusion criteria are applied to the source data and a power and sample size calculation table for feasibility counts, if relevant. The exact contents of this attrition table can vary depending on the type of calculation. A glossary of terms helps avoid misinterpretations, which over time will support standardisation of the terminology used by investigators and reviewers (appendix table 8). A list of abbreviations is also included (appendix table 9).

The template tables should always be accompanied by appendices that contain specific clinical code lists (eg, International Classification of Diseases-Clinical Modification, 9th revision; Current Procedural Terminology; READ codes) used to define study entry criteria or exposure, inclusion or exclusion, confounders, outcomes, and structured to be machine read or writeable. These tables can also be accompanied, if relevant, by additional appendices that detail decisions made during the conversion process from source data to a common data model, which provide more information about data linkages or other data processing steps, as well as appendices that contain or provide links to code used to create and analyse the study population. The focus group findings are summarised in appendix 2, arranged by main themes regarding the value, challenges, and usability for international RWE stakeholders.

## STaRT-RWE example library from published studies

We developed a library of STaRT-RWE examples featuring common use cases involving different study designs, sources of data, and more complex algorithms to define key study parameters. These four case studies, based on published studies, aim to increase usability and facilitate adoption by providing tangible examples of how the template would be populated for different study designs and data sources. These examples are easily modifiable to fit similar use cases and include a comparative effectiveness cohort study, a predictive outcome modelling study using data from linked claims and electronic health records, a study of drug treatment safety in pregnancy, and a study using a self-controlled design (appendix 3). An example of structured appendices containing code lists that are machine readable or writable to accompany the STaRT-RWE study implementation template tables is provided (appendix 3).

## Discussion

A key hurdle for RWE studies on the use, effectiveness, and safety of medical products to be considered by decision makers is the perceived ambiguity and lack of detail on complex design and analysis choices. Through a public-private collaboration with broad, international stakeholder input, we have developed a structured template for planning and reporting on RWE study implementation. The template is intended to aid study design, conduct, and review in a way that adds value for researchers, sponsors, reviewers, and decision makers. STaRT-RWE is not a reporting checklist but can complement existing checklists by providing guidance and a common structure to help research teams be clear and comprehensive when planning and communicating critical details of intended scientific decisions. By using tabular and visual formats, the template (unlike a checklist) minimises ambiguous prose and potential for misinterpretation.

STaRT-RWE is compatible with multiple study designs and RWD sources. It accords with an earlier recommendation by a joint ISPE-ISPOR consensus document on specific parameters that are necessary to make RWE research reproducible^15^ as well as published bias assessment tools.^29^ ^30^ ^31^ The study implementation template aims to be used in conjunction with unstructured prose based introductions and discussions detailing background and rationale, investigator’s interpretation of results, and noted strengths and limitations of the study. On completion of a study, the STaRT-RWE tables and figure detailing the study methods should be accompanied by appropriate tables and figures to communicate the results.

The planning and reporting of clinical trials are standardised. STaRT-RWE parallels the trial setting by providing a structured way to communicate complex RWE study design and analysis choices. The goal is not to standardise how people do the science of RWE, but rather to provide a framework for researchers to work through the details of design and analysis with a common understanding of the decisions that are made.

Clinical research studies are currently expected to provide a CONSORT diagram to describe how the study population was derived. We hope that RWE studies will include a “figure 1” for the study design diagram and an “appendix 1” for structured study parameter tables to clearly communicate the details of how the population, exposure, outcomes, and covariates being studied were derived from longitudinal streams of healthcare data not collected for research.

The structured template we have developed is operationally focused, tabular, and visual, minimising prose and reducing the potential for misinterpretation. The template’s study parameter tables and figure are designed to be flexible enough to incorporate into existing processes for protocol development and reporting. We expect that after the initial learning curve and refinements, use of the template would become routine with practice. Furthermore, software solutions can be developed to read input or output study tables, design diagrams, and appendices with a common structure.^3^ ^8^ ^34^ ^35^ ^36^ ^37^ Given the trend towards registration of hypothesis confirming RWE studies,^28^ STaRT-RWE or a subset of core elements could be integrated with RWE study registration sites to simplify the registration process and improve standardised searches. However, many studies aim to raise hypotheses that need to be evaluated rather than confirm them. Registration might be less imperative in such settings. Furthermore, we recognise that other ways of communicating and implementing shared protocols already exist. Investigators who are unable to use the template should not be discouraged from publishing and reporting their observations.

Sharing data and code allows computational reproduction. STaRT-RWE facilitates replicability and validity assessment. Sharing of data and code should be encouraged when possible to allow computational reproduction. However, for research using routinely collected electronic healthcare data, public sharing of source data or data derivatives are typically not permissible. Furthermore, providing code does not substitute for clear communication of the intended study implementation parameters. A concern raised by stakeholders is that a narrow focus on computational reproducibility could encourage investigative teams to be less careful in communication of methods. The detailed logic flow contained in the template will provide more insight into the intended scientific design parameters than can be found in code by most readers. Additionally, code to create analytical study populations from source data are not readily transferable between data sources that have different database schemes whereas the information in the template is transferable. By providing the detailed study recipe that most reviewers would not be able to parse from code, the template facilitates validity assessment, independent reproducibility in the same data source, and replication in different data sources.

### Limitations

The STaRT-RWE template has a few limitations. Firstly, while the template is designed to be flexible, the structure imposed in the tables might be an awkward fit for some use cases. Depending on the study, only a subset of tables in STaRT-RWE might be relevant. Secondly, use of the study implementation template to guide the planning of a study and provide clarity about scientific decisions does not guarantee that those decisions will result in unbiased findings. However, appropriate interpretation of study findings will be greatly facilitated if reviewers see unambiguous information on how those findings were derived, and what strategies were used to mitigate potential biases. 

Thirdly, the template focuses primarily on study implementation decisions. Although the template includes a section about the data sources, the fields in the template do not capture all of the information needed to assess whether the data are fit-for-purpose. The fitness of data sources should be clearly documented, including a description of the available data fields and completeness of data capture (eg, closed *v* open system, inpatient *v* outpatient, primary *v* specialty care). They should also be accompanied by clear data provenance documenting transformations performed on the data streams used to create the research database. However, this level of metadata about the data source was not included in the STaRT-RWE template because we thought it would be more efficient for documentation to be created, maintained, and made publicly available as a citable resource for each RWD source, rather than having the detailed data source documentation repeated for every RWE study that uses the data source.

### Dissemination plan

To harmonise protocol and reporting standards for regulatory submissions and coverage decisions,^26^ more work will be necessary, including dissemination of STaRT-RWE and discussion with regulators, health technology assessment agencies, and other relevant stakeholder organisations; development of training modules; use of the template for protocols posted in study registries such as EU-PASS or other governance processes; coordination with study registration sites to enable electronic uploading of the completed template when pre-registering a RWE study; and engagement of medical journal editors. Collection of feedback from broad stakeholder groups with practical experience of the template will be critical to inform updates. Multiple workstreams have been initiated to implement these next steps, support researcher training, and integrate STaRT-RWE with existing processes.^25^ ^26^ ^38^

### Conclusion

STaRT-RWE is designed to reduce misinterpretation and create clear expectations regarding communication of how RWE is generated—expectations about which study design and analysis details should be reported as well as how and where that information should appear. The study implementation template is not a substitute for a well trained pharmacoepidemiologist or outcomes researcher, and unambiguous communication of study implementation is not necessarily equivalent to highly valid, rigorous study methodology. However, the availability of a baseline level of information about study methodology in a consistent structure would improve clarity regarding study implementation decisions between study investigators and decision makers. This information would increase healthcare decision makers’ ability to effectively evaluate RWE study validity.

## Web extra

Extra material supplied by authors

Appendix 1: STaRT-RWE template tables and figure

Appendix 2: Summary of focus group feedback

Appendix 3: Summary of the library of STaRT-RWE examples

## Notes

Ethical approval: This study was approved by the institutional review board of Brigham and Women’s Hospital.

Data sharing: The STaRT-RWE template and examples will be publicly available. Additional details of the focus groups beyond what is summarised in the appendices are available on request.

The corresponding author affirms that the manuscript is an honest, accurate, and transparent account of the study being reported; that no important aspects of the study have been omitted; and that any discrepancies from the study as planned have been explained.

Patient and public involvement: We sought feedback on the usability, value, and challenges of using the structured study implementation template via focus groups with international leaders involved in generating or reviewing RWE for decision making. Focus group participants included regulators, health technology assessors, industry, journal editors, payers, and leaders of large distributed data networks. After revising the template based on their suggestions, we sought feedback from ISPE and ISPOR membership and received endorsement from both international societies.

Dissemination to participants and related patient and public communities: We intend to disseminate STaRT-RWE to international stakeholder groups involved with generating or using real world evidence through publication, conference presentations, as well as multistakeholder efforts focused on increasing transparency of real world evidence studies. These efforts will involve collaboration and coordination with ISPE, ISPOR, the International Council of Harmonization workgroup on harmonizing technical protocols, Transparency Initiative, clinicaltrials.gov, Open Science Framework, European Medicines Agency’s Big Data Task Force, EU Electronic Register of Post-Authorisation Studies, European Network of Health Technology Assessment, the EQUATOR network, and others.

Provenance and peer review: Not commissioned; externally peer reviewed.
