# Supplementary material for: STaRT-RWE: structured template for planning and reporting on the implementation of real world evidence studies
Source: BMJ. 2021 Jan 12;372:m4856. doi: 10.1136/bmj.m4856 (PMC8489282; doi:10.1136/bmj.m4856)
Supplement: Supplementary file 1 — Appendix 1: STaRT-RWE template tables and figure [file wans059917.ww1.pdf]

Structured Template and Reporting Tool for Real World Evidence (STaRT-RWE)

TABLE OF CONTENTS

Table 1. Administrative Information
Table 2. Version History
Figure 1. Design Diagram
Table 3. Summary of Analytic Study Population
    A. Meta-data about data source and software
    B. Index Date (day 0) defining criterion
    C. Inclusion Criteria
    D. Exclusion Criteria
    E. Predefined Covariates
    F. Empirically Defined Covariates
    G. Outcome
    H. Follow up
Table 4. Analysis Specification
Table 5. Sensitivity Analyses
Table 6. Attrition Table
Table 7. Power and Sample Size Calculation
Table 8. Glossary of Terminology
Table 9. Abbreviations

TABLE 1. ADMINISTRATIVE INFORMATION

Instructions: Fill in the yellow highlighted sections where applicable.

**Protocol Title:**			
**Objective:**<br>*Include PICOTS (Patient, Intervention, Comparator, Outcome, Time-Horizon, Setting)*			
	**Primary:**		
	**Secondary:**		
**Protocol registration:**	**Registration identifier**	**Registration date**	**Registration site**
**Protocol version:**	**Version number**	**Version date**	
**Protocol Contributors:**	**Name**	**Role**	**Affiliation**
**Funding:**	**Grant identifier**	**Source**	
**Data Use Agreement (DUA)**	**DUA identifier**	**Data provider**	**Data provider contact for data use agreements**
**Human Subjects/Ethics Approval**	**Submission identifier**	**Date of approval**	**Name of human subjects/ethics approval committee**

TABLE 2. VERSION HISTORY

Instructions: Fill in the yellow highlighted sections to log changes and rationale for making changes made to the prior version of the protocol.

Version date	Version number	Change log	Rationale for change

FIGURE 1. DESIGN DIAGRAM

Instructions: Create design diagram using the framework outlined in Schneeweiss et al. Graphical Depiction of Longitudinal Study Designs in Health Care Databases. Ann Intern Med. 2019;170:398–406. The diagram can be created using power point templates or other software program of choice. It is intended to be read from top to bottom, reflecting the order of operations to create an analytic cohort from a source longitudinal healthcare database. Temporality of assessment windows are clearly shown relative to the cohort entry (“index”) date, which is considered day 0. Bracketed number ranges denote the inclusive time windows for washout, inclusion/exclusion, and covariate assessment windows as well as follow up. Whether or not day 0 is included in an assessment window can also be visually distinguished by whether it overlaps the vertical arrow representing the cohort entry date

The diagram may include footnotes specifying the inclusion/exclusion criteria, covariates, and censoring criteria relevant to each assessment window.

A. Meta-data about data source and software

This section records the calendar time range used to ascertain cohort entry (index date), as well as the calendar time range of data available for pre-index assessment windows and post-index follow up (study period). The data source name and version are identified, as well as any sampling criteria applied (for example, the data cut only includes patients with a diagnosis of diabetes). If there is data linkage involved, provide a citation or an appendix with description of the linkage (how, performance characteristics)

	Data Source 1	Data Source 2	Data Source 3	Data Source 4
**Data Source(s):**				
**Study Period:**				
**Eligible Cohort Entry Period:**				
**Data Extraction Date/Version:**				
**Data sampling/extraction criteria:**				
**Type(s) of data:**				
**Data linkage:**				
**Data conversion:**				
**Software to create study population:**				

Code algorithms for cohort entry date are provided in Appendix A, study entry criteria in Appendix B, covariates in Appendix C and D, outcomes in Appendix E (a * in code algorithm indicates use of a wildcard)

All temporal windows anchored on study population entry date (Day 0) unless otherwise specified.

( ) represent open intervals that do not include the end points

[ ] represent closed intervals that do include the end points

[1] Please enter all that apply. Valid entries: IP = inpatient, OP = outpatient, ED = emergency department, any, other, n/a = not applicable. See Appendix E for details on how care setting is defined

[2] Specify whether a diagnosis code is required to be in the primary position (main reason for encounter)

TABLE 3. SUMMARY SPECIFICATION FOR ANALYTIC STUDY POPULATION

Instructions: Fill in the yellow highlighted sections.

B. Index Date (day 0) defining criterion

The criterion that define the date of entry to the cohort(s) is specified in this section. There should be one row for each unique definition of study population entry. If the study is descriptive, there may only be one row filled out. An active comparator study may have 2 rows, one for the exposure of interest and one for the comparator.

Check the pre-specified box if the exclusion criterion was specified before beginning data analyses, check the varied for sensitivity box if it was modified as part of sensitivity analyses. Specify the source of algorithms to define study entry criteria.

Study population name(s)	Day 0 Description	Number of entries	Type of entry	Washout window	Care Setting[1]	Code Type	Diagnosis position[2]	Incident with respect to…	Pre-specified	Varied for sensitivity	Source of algorithm

C. Inclusion Criteria

Describe what the criterion is conceptually. Specify the order of application of the inclusion criteria is relative to selection of the index date (day 0) for study entry. For example, specify “after selection of index date” if you plan to 1) select the index date based on first time the study entry defining criterion is met in the study period, 2) then apply inclusion-exclusion criteria, 3) keep the selected index date for study entry if all inclusion-exclusion criteria are met. Alternatively, you can specify “before selection of index date” if you plan to 1) identify all potential index dates meeting the study entry criterion, 2) apply inclusion-exclusion criteria, 3) select one or more of the study entry dates that meet all inclusion-exclusion criteria. Define the assessment window relative to the index date, whether there are restrictions on care setting or diagnosis position in the algorithm to define each inclusion criterion and specify which study populations (defined in Table 3B) the criterion is applied to.

Defining “observable” patient time in the healthcare data source is almost always required as an inclusion criterion. When using administrative claims data, this can be measured with dates of enrollment in insurance coverage, with or without bridging of short gaps in enrollment. When using electronic health record data, defining observable patient time may require making some strong assumptions. For example, assuming that patient encounters are always observable, that patients are observable between the first and last recorded encounter in the record, that patients are observable for X days before and after any recorded encounter, etc. Alternatively, one could specify inclusion based on algorithms to measure “loyalty” to a healthcare provider or EHR system.

Check the pre-specified box if the exclusion criterion was specified before beginning data analyses, check the varied for sensitivity box if it was modified as part of sensitivity analyses. Specify the source of algorithms to define inclusion criteria.

Criterion	Details	Order of application	Assessment window	Care Settings[1]	Code Type	Diagnosis position[2]	Applied to study populations:	Pre-specified	Varied for sensitivity	Source for algorithm
…			…	…			…			…

TABLE 3. SUMMARY SPECIFICATION FOR ANALYTIC STUDY POPULATION
Instructions: Fill in the yellow highlighted sections.

D. Exclusion Criteria
Describe what the criterion is conceptually. Specify the order of application of the exclusion criteria is relative to selection of the index date (day 0) for study entry. Define the assessment window relative to the index date, whether there are restrictions on care setting or diagnosis position in the algorithm to define each exclusion criterion and specify which study populations (defined in Table 3B) the criterion is applied to.

Check the pre-specified box if the exclusion criterion was specified before beginning data analyses, check the varied for sensitivity box if it was modified as part of sensitivity analyses. Specify the source of algorithms to define exclusion criteria.

Criterion	Details	Order of application	Assessment window	Care Settings[1]	Code Type	Diagnosis position[2]	Applied to study populations:	Pre-specified	Varied for sensitivity	Source for algorithm

TABLE 3. SUMMARY SPECIFICATION FOR ANALYTIC STUDY POPULATION
Instructions: Fill in the yellow highlighted sections.

E. Predefined Covariates
Define the covariate conceptually, with accompanying details as necessary. Specify which planned analyses adjust for the covariate, and how it is specified in the analysis (e.g. continuous, categorical, binary). Define the assessment window relative to the index date (day 0), whether there are restrictions on care setting or diagnosis position in the algorithm, and which study populations defined in Table 3B the covariate is measured for. Specify the source of algorithms to define covariates.

Check the pre-specified box if the covariate was specified before beginning data analyses, check the varied for sensitivity box if it was modified as part of sensitivity analyses. Specify the source of algorithms to define covariates.

Characteristic	Details	Type of variable	Assessment window	Care Settings[1]	Code Type	Diagnosis position[2]	Applied to study populations:	Pre-specified	Varied for sensitivity	Source for algorithm

Code algorithms for cohort entry date are provided in Appendix A, study entry criteria in Appendix B, covariates in Appendix C and D, outcomes in Appendix E (a * in code algorithm indicates use of a wildcard)
All temporal windows anchored on study population entry date (Day 0) unless otherwise specified.
( ) represent open intervals that do not include the end points
[ ] represent closed intervals that do include the end points
[1] Please enter all that apply. Valid entries: IP = inpatient, OP = outpatient, ED = emergency department, any, other, n/a = not applicable. See Appendix E for details on how care setting is defined
[2] Specify whether a diagnosis code is required to be in the primary position (main reason for encounter)

TABLE 3. SUMMARY SPECIFICATION FOR ANALYTIC STUDY POPULATION

Instructions: Fill in the yellow highlighted sections.

F. Empirically Defined Covariates

Empirical identification of covariates to use in confounding control may not be relevant to all study populations or analyses, however if such methods are used, this section includes fields to describe what the algorithm for covariate identification is, as well as specification of the settings or parameters used to empirically identify covariates. In this section, specify the assessment window relative to the index date (day 0), which analyses adjust for empirically identified covariates, how the covariates are specified in a model, whether there are restrictions on care setting or diagnosis position, and which study populations (defined in section 3B) to measure the empirical covariates.

Check the pre-specified box if the empirical covariate selection parameters were specified before beginning data analyses, check the varied for sensitivity box if the parameters were modified as part of sensitivity analyses. Specify the source for the method and/or software used for empirically defined covariates.

Algorithm	Type of variable	Assessment window	Care Settings[1]	Code Type	Diagnosis position[2]	Applied to study populations:	Pre-specified	Varied for sensitivity	Source/code for algorithm

TABLE 3. SUMMARY SPECIFICATION FOR ANALYTIC STUDY POPULATION
Instructions: Fill in the yellow highlighted sections.

G. Outcome
Define the outcome conceptually and whether it is the primary outcome of interest. Specify whether the type of outcome is incident (if so, there is a field to specify the washout window to define "incident" occurrences), prevalent or other. Specify whether there are restrictions on care setting or diagnosis position, and which groups or analyses the outcome is measured for. If there are measurement characteristics for the outcome algorithm (e.g. PPV, sensitivity, specificity) from publications, or from outcome validation within the study population (e.g., medical record review), provide this information.

Check the pre-specified box if the outcome parameters were specified before beginning data analyses, check the varied for sensitivity box if the parameters were modified as part of sensitivity analyses. Specify the source of algorithms to define outcomes.

Outcome name	Outcome measurement characteristics	Primary outcome?	Type of outcome	Washout window	Care Settings[1]	Code Category	Diagnosis position[2]	Applied to study populations:	Pre-specified	Varied for sensitivity	Source of algorithm

TABLE 3. SUMMARY SPECIFICATION FOR ANALYTIC STUDY POPULATION

Instructions: Fill in the yellow highlighted sections.

H. Follow up

Specify when follow up begins relative to the index date (day 0) and select each criterion that is used to end follow up.

Check the pre-specified box if the outcome parameters were specified before beginning data analyses, check the varied for sensitivity box if the parameters were modified as part of sensitivity analyses.

			Pre-specified	Varied for sensitivity
**Begins**				
**Ends**	**Select all that apply**	**Specify**		
**Date of Outcome**				
**Date of Death**				
**Date of Disenrollment**				
**Day X following index date** *(specify day)*				
**End of study period** (specify date)				
**End of exposure** *(specify operational details, e.g. stockpiling algorithm, grace period)*		**Stockpiling algorithm:** **Grace period:**		
**Date of add to/switch from exposure** *(specify algorithm)*				
**Other** *(specify)*				

Code algorithms for cohort entry date are provided in Appendix A, study entry criteria in Appendix B, covariates in Appendix C and D, outcomes in Appendix E (a * in code algorithm indicates use of a wildcard)

All temporal windows anchored on study population entry date (Day 0) unless otherwise specified.

( ) represent open intervals that do not include the end points

[ ] represent closed intervals that do include the end points

¹ Please enter all that apply. Valid entries: IP = inpatient, OP = outpatient, ED = emergency department, any, other, n/a = not applicable. See Appendix E for details on how care setting is defined

TABLE 4 ANALYSIS SPECIFICATIONS
Instructions: Fill in the yellow highlighted sections.

Specify the study populations (defined in 3B) included in the analysis, the outcome being evaluated, the software that is used, the type of confounding adjustment, missing data methods and subgroup analyses.

	Primary	Secondary 1	Secondary 2
**Hypothesis:**<br><br>**Study population(s)**<br>**Outcome:**<br><br>**Software:**<br>**Model(s):**			
**Confounding adjustment method**<br>***(check all that apply and provide details where requested)***<br>**Bivariate**<br>**Multivariable**<br>**Propensity score matching**<br>***(specify matching algorithm, ratio and caliper)***<br>**Propensity score weighting**<br>***(specify weight formula, trimming, truncation decisions)***<br>**Propensity score stratification**<br>***(specify strata definition)***<br>**Other**<br>***(specify details)***			
**Missing data methods**<br>**(check all that apply, provide relevant details)**<br>Missing indicators<br>Complete case<br>Last value carried forward<br>Multiple imputation (specify variables)<br>Other (please specify)…			
**Subgroup Analysis**			

TABLE 5 SENSITIVITY ANALYSES
Instructions: Fill in the yellow highlighted sections.

	What is the parameter being varied? (be clear what it is changing from)	Why? (What do you expect to learn?)	Strengths of the sensitivity analysis compared to the primary?	Weaknesses of the sensitivity analysis compared to the primary?
**Sensitivity Analysis 1**				
**Sensitivity Analysis 2**				
**Sensitivity Analysis 3**				

TABLE 5. ATTRITION TABLE

Instructions: Fill in the yellow highlighted sections. Show the number of patients remaining after applying each inclusion/exclusion criterion, sequentially.

	**Drug A**		**Drug B**	
	**Excluded Patients**	**Remaining Patients**	**Excluded Patients**	**Remaining Patients**

Drug A initiators
N =

Drug B initiators
N =

TABLE 7. POWER AND SAMPLE SIZE CALCULATION

Instructions: Fill in the yellow highlighted sections.
Specify the software used, what is being calculated (e.g. power, sample size, detectable difference), the population and statistical assumptions for the calculations. For each parameter assumption, specify the primary assumption and the range considered. Specify the sources used to select the estimated population parameters. The power or sample size calculations across the range of assumed parameter values may be displayed in tabular or visual form as needed.

The template contains assumptions to calculate power for a comparison of 2 proportions, however the entries for the population assumptions and statistical parameters should be modified to reflect those that are relevant for the calculation used by the investigators.

**Software:**			
**Calculate:**			
**Population assumptions**	**Primary**	**Range**	**Source for estimated parameters**
**Statistical parameters:**			

TABLE 8 GLOSSARY

Term	Definition
Confounder	Variable other than the exposure of interest or outcome under investigation that is 1) associated with exposure, 2) a risk factor for the outcome, and 3) not on the causal pathway between the exposure and the outcome. A confounder can artificially inflate or reduce the magnitude of association between an exposure and outcome.
Covariates	Variables that are neither exposure nor outcome of interest, but are measured to describe a population or because they may be a confounder to account for in analyses
Data Extraction Date	The date when the data were extracted from the dynamic healthcare database
Days Supplied	Number of days supplied for a dispensed prescription
Eligible cohort entry period	Calendar time frame during which cohort entry dates are identified
Empirically defined covariates	Covariates that are not prespecified by the investigator. The selection of these covariates is based on applying algorithms to the data. The algorithms for covariate selection may be tuned by investigator specified parameters.
Observable Time	For insurance claims data, this may refer to periods of enrollment in medical and/or drug plans. For electronic health record databases, this may refer to algorithms designed to identify patients whose healthcare contacts are likely to be covered within the healthcare system.
Opbservable Time Gap	Maximum number of days allowed between two consecutive observable time windows to still be considered “continuously observable”.
Follow up window	The interval during which occurrence of the outcome of interest in the study population will be included in the analysis.
Grace Window	Number of days added to days supply to allow for non-adherence or account for the hypothesized biologic exposure risk window. Operationally, this could be defined as the number of extra days added to the end of a days supply to extend time counted as "exposed". This grace may bridge the gap between dispensations where the days supply dispensed does not cover all days until the refill.
Index Date	The date when subjects enter the study population (cohort entry date, outcome event date). It is defined based on events in the patient's longitudinal timeline, other windows are defined relative to the index date.
Assessment Window	Interval during which a patient is required to have evidence of a pre-existing condition (diagnosis/procedure/drug dispensing). May be used for washout of exposure or outcome, exclusion assessment or covariate assessment.
Predefined Covariates	Covariates that are prespecified and defined by the investigator in the protocol.
Principal Diagnosis	Diagnosis or condition established to be chiefly responsible for admission of the patient to the hospital.
Source Data Range	The calendar time range covered by a data source that is available
Stockpiling Algorithm	Algorithm defining how early refills are handled when determining length of exposure follow up
Study Period	Calendar time interval of data available for study, including pre-index date assessment windows and post-index follow up
Treatment Episode	Continuous period of exposure defined using by dispensation date + days supply and applying stockpiling algorithms and/or grace windows
Washout Window	Minimum number of days a patient is required to have no evidence of prior exposure and/or outcome
Wildcard	Symbol used to represent any single alphanumeric digit in code algorithms. For example 410.*1, where * is the wildcard.

ABBREVIATIONS

RX = drug prescription/dispensation
DX = diagnosis
PX = procedure
LB = laboratory
NDC = national drug code
ICD-9-CM = International Classification of Diseases - Clinical Modification 9th revision (2012)
ICD-10-CM = ICD-CM 10th revision
ICD-11-CM = ICD-CM 11th revision
CPT = Current Procedural Terminology
HCPCS = Healthcare Common Procedure Coding System
LOINC = Logical Observation Identifiers Names and Codes
IP = inpatient
OP = outpatient
ED = emergency department
n/a = not applicable
