# Supplementary material for: STaRT-RWE: structured template for planning and reporting on the implementation of real world evidence studies
Source: BMJ. 2021 Jan 12;372:m4856. doi: 10.1136/bmj.m4856 (PMC8489282; doi:10.1136/bmj.m4856)
Supplement: Supplementary file 2 — Appendix 2: Summary of focus group feedback [file wans059917.ww2.pdf]

Appendix 2 Focus group summary of feedback

Value

1. Assists regulatory and other healthcare policy decision-making by facilitating discussion, minimizing miscommunication and assumptions

   Stakeholders in the focus groups felt that STaRT-RWE could improve clarity of communication about the methods implemented to generate RWE between researchers and decision-makers as well as between multi-disciplinary study team members. Specifically, in communications between investigators and regulators, the template was perceived by regulators as a tool that could help investigators clearly explain what they are planning to do before beginning a study and help them work together to come to agreement on a protocol.

   Several academic and industry stakeholders expressed that prose-based protocols often lack important details and in multi-disciplinary study teams, there can be miscommunication and missed assumptions when protocols prepared by an investigator are implemented by a programmer or data scientist. They saw potential for the tables and figure of STaRT-RWE to enhance their existing processes by making explicit the intended decisions that will have to be made at some point during study implementation, leaving less room for different interpretation of protocols. There was universal enthusiasm for the graphical framework for representing study design, with multiple stakeholders reporting that their organizations already have or are planning to use the graphical depiction of design framework to enhance communication.

2. Facilitates evidence synthesis

   Regulators and other healthcare decision-makers felt that if STaRT-RWE would be very helpful when there are multiple RWE studies to review in parallel because non-uniformity in reporting makes it difficult to put research in context and compare apples to apples. Furthermore, by providing a framework that encourages provision of comprehensive reporting of key design, analysis and other methodological decisions, the tool would make it easier to use bias assessment tools such as ROBINS-I[21], where stakeholders reported that their reviewer teams often have to say “unclear” to many checklist items when trying to assess validity.

3. Useful teaching purpose - Makes the researcher think systematically and not forget something

   Several stakeholders mentioned the utility of STaRT-RWE as a teaching or training tool; that by systematically walking researchers through items that are important to think about when planning a RWE study, it would improve study design. One stakeholder put it succinctly, “better planning makes better studies.” Stakeholders also felt that the template addressed a need for standardization in protocol writing.

4. Comprehensive yet flexible for different users/audience

   The flexibility of the structured tables to be used for many different purposes was viewed as a beneficial feature, where relevant tables and figures could be presented for relevant audiences. For example, stakeholders felt the whole set would likely be relevant for

communications with regulatory agencies, while a subset could be included as appendices in manuscripts or reports. Others felt the template tables could form the basis of a public protocol for study registration or internal communications with a data scientist/programmer. Inclusion of machine-readable appendices containing code algorithms to define key study parameters was seen as a useful feature because it allowed them to be directly used in statistical programming and removed a step for potential miscommunication.

5. Promotes harmonization in protocol development and reporting - parallels structured regulatory environment for trials

   Stakeholders brought up parallel efforts for clinical trials designed to provide more transparency and harmonization for communication about trial methods and study process including the TransCelerate[24] collaboration, the National Institute's of Health e-protocol writing tool for trials[25] and the FDA's highly structured, and regulated environment for trials. Stakeholders felt that if something like STaRT-RWE's structured tables became an accepted common standard for communicating RWE study methods, their organizations would be able to develop macros, software, and other infrastructure and processes to fit that standard, thereby increasing efficiency on their end.

6. Advances replicability

   Participants in focus groups were in agreement about the comprehensiveness of the elements in STaRT-RWE and recognized that communication of such details would greatly facilitate replicability of RWE studies in the same data or different data.

Challenges

1. More work for researchers

   Stakeholders across the board felt that widespread use of STaRT-RWE would benefit reviewers and decision-makers but that the additional burden for researchers was a major challenge toward adoption. For some participants, learning that if scientific decisions had already been settled, then manually filling out the template was expected to take no more than a few hours was reassuring. For others, a few hours was viewed as an inordinate burden when scaled up to the sheer number of studies conducted by their organization and the many layers of oversight and review for each.

   Because the template guides research teams to think about and explicitly report key scientific decisions that are made during implementation, requiring additional effort for researchers to use STaRT-RWE was also seen as a positive attribute by some stakeholders, with several saying that it was "the price of being an adult" or that having some barriers may be a good thing, because it would raise the level of expertise needed to do a RWE study.

2. Need incentives

   Many reported that it would take time and incentives for their organizations to overcome resistance to adjusting existing processes and standard operating procedures. For example, being able to use the template to address multiple governance processes such as study registration, ethics review, and/or publication. Stakeholders mentioned inertia

as another challenge to using STaRT-RWE, that change would happen slowly, if at all, unless there were regulatory implications. Several stakeholders stated that their adoption would be spurred by the knowledge that the information they provide in the template would be used by someone, whether it be for regulatory or other purposes. Therefore, regulatory discussion, recommendations and requirements on RWE study protocols will be critical to shaping future adoption and trickle-down effects. With respect to inertia, stakeholders recommended aiming to teach students and trainees to think and use this structured way of developing RWE study protocols and reporting. One journal editor broached the radical idea of using a template table to communicate study methodology rather than prose based methods.

3. What is minimum necessary? Which parts for which purposes?

   In reference to concerns about increased burden, stakeholders questioned whether all studies would need such a high level of rigor in reporting. Stakeholders raised the point that different users have different needs – a data analyst needs the appendices whereas a reviewer doesn't necessarily need to see those. They generally acknowledged that if a study was expected to be highly influential for regulatory decision-making, healthcare policy or practice, then the level of detail in STaRT-RWE would be called for. While the flexibility of the template was seen as a useful feature, uncertainty about which parts to use for which purposes was expected to impede adoption. This uncertainty could be addressed by end users and gate keepers (i.e. regulators, health technology assessors, payers, journal editors) setting their own thresholds and being clear about which parts of the template they want to see as part of RWE study reporting.

4. Need centralized site designed for RWE to post protocols

   Some stakeholders felt that it was important to have a standardized protocol template like STaRT-RWE and a central location for storing this data. A few mentioned the initiation of scattered and uncoordinated sites by various organizations for registering and depositing protocols as evidence of the increasing focus on transparency, however, the lack of a centralized site or process for posting of RWE protocols was viewed as a challenge.

5. Difficult to see how more complicated studies and sensitivity analyses would fit with the template.

   Some stakeholders had difficulty seeing how studies with numerous sensitivity analyses or complicated algorithms to define study parameters would fit within the templates structured framework. Others raised concerns that the template was too focused on the cohort design or too US-centric. They felt there was a need to develop a library of STaRT-RWE examples featuring common use cases involving different study designs, sources of data, and more complex algorithms to define key study parameters to demonstrate usability.

Main usability issues

1. Need for library of examples to facilitate usability

   Heeding the call from stakeholders, we prepared a small library of STaRT-RWE examples to increase usability and facilitate adoption (online appendices). These use

cases provide tangible examples of structured tables and figures for different study designs and data sources and are easily modifiable to fit similar use cases. The 4 examples include 1) a comparative effectiveness cohort study, 2) predictive outcome modeling in linked claims-electronic health record data, 3) safety of medications in pregnancy, and 4) a self-controlled design.

2. Add a table to summarize sensitivity analyses

   The version of STaRT-RWE reviewed by focus group participants did not include Appendix Table 5. This table was added to address concerns that it would be unwieldy to fill out or review an entire set of tables for each sensitivity analysis, and that a critical piece of sensitivity analyses to the decision-maker was understanding how the alternative analysis could inform understanding.

3. Desire for software to auto-create design diagram, populate protocol

   Stakeholders were enthusiastic about the idea of an online structured template with back-end analytics to streamline the conduct and reporting of RWE studies.

   Currently, members of the investigative team are developing an open-source R Shiny App that can create design diagrams to scale. The FDA's Sentinel Initiative has developed a visual query builder which can populate the structured inputs necessary to create and analyze cohorts with their routine query tools. If such structured tables and design diagram as contained within STaRT-RWE were to become a common standard, other software developed for conducting RWE studies, such as the Aetion Platform or OHDSI's tools, could be adapted to use structured tables and a design diagram as inputs and/or produce them as outputs, thus greatly increasing workflow efficiency as well as communication of scientific decisions.
