# Supplementary material for: STaRT-RWE: structured template for planning and reporting on the implementation of real world evidence studies
Source: BMJ. 2021 Jan 12;372:m4856. doi: 10.1136/bmj.m4856 (PMC8489282; doi:10.1136/bmj.m4856)
Supplement: Supplementary file 3 — Appendix 3: Summary of the library of STaRT-RWE examples [file wans059917.ww3.pdf]

Appendix 3. Summary of the library of STaRT-RWE examples

The examples provided in the appendices are abbreviated and modified from existing studies to highlight features of the template. These examples include:

1. **Propensity score matched comparative effectiveness cohort study**
    - Empagliflozin versus DPP-4 Inhibitor on risk of 3P MACE in IBM MarketScan Research Database, Optum Clinformatics, and Medicare claims data[1]
    - This example of a new-user, active comparator cohort study includes a more complex inclusion criterion algorithm requiring Boolean logic
2. **Predictive outcome modeling with EHR data**
    - Prediction of ejection fraction class in patients with heart failure[2]
    - This example is a cohort study focusing on prediction/classification of outcomes that are not based on diagnosis/procedure codes in a claims-EHR linked data source
3. **Safety of medications in pregnancy**
    - Risk of oral clefts associated with topiramate exposure in the first trimester of pregnancy using propensity score fine-stratification to adjust for confounding[3]
    - This example uses a mother-infant linked claims data source
4. **Alternative to cohort study design**
    - Measles-mumps-rubella vaccination and risk of seizure[4,5]
    - This example uses Danish registry data and a self-controlled risk interval design

All STaRT-RWE tables, figure and detailed code appendices are populated for the first example, to demonstrate how these tables would be filled out for a straightforward propensity score matched comparative effectiveness study. The design diagram, study parameter and analysis tables are populated for only the primary analysis of the remaining examples, to demonstrate how different design and analysis plans could be reported with STaRT-RWE.

To increase clarity about which sections are intended to be filled in by the research team, the relevant fields where it is the researcher's responsibility to report a study parameter are highlighted in yellow.

Each of these 4 STaRT-RWE examples can be accessed at Harvard Dataverse:
https://dataverse.harvard.edu/dataset.xhtml?persistentId=doi:10.7910/DVN/6R1KCA

The source studies cited below were used to populate STaRT-RWE examples:


1. Franklin JM.; Pawar AS, S. Replication of the EMPAREG Diabetes Trial in Healthcare Claims. US National Library of Medicine. Study Registration Record Web site. https://clinicaltrials.gov/ct2/show/NCT04215536?id=NCT04215536&draw=2&rank=1&load=cart. Published 2019. Accessed March 4 2020.
2. Desai Rishi J, Lin Kueiyu J, Patorno E, et al. Development and Preliminary Validation of a Medicare Claims–Based Model to Predict Left Ventricular Ejection Fraction Class in Patients With Heart Failure. *Circulation: Cardiovascular Quality and Outcomes.* 2018;11(12):e004700.
3. Hernandez-Diaz S, Huybrechts KF, Desai RJ, et al. Topiramate use early in pregnancy and the risk of oral clefts: A pregnancy cohort study. *Neurology.* 2018;90(4):e342-e351.
4. Vestergaard M, Hviid A, Madsen KM, et al. MMR Vaccination and Febrile SeizuresEvaluation of Susceptible Subgroups and Long-term Prognosis. *Jama.* 2004;292(3):351-357.

5. Klein NP, Fireman B, Yih WK, et al. Measles-mumps-rubella-varicella combination vaccine and the risk of febrile seizures. *Pediatrics.* 2010;126(1):e1-8.
